# Effects of Tai Chi on Sleep Quality as Well as Depression and Anxiety in Insomnia Patients: A Meta-Analysis of Randomized Controlled Trials

**DOI:** 10.3390/ijerph20043074

**Published:** 2023-02-09

**Authors:** Min Yang, Jiaqi Yang, Mingjun Gong, Rui Luo, Qinqin Lin, Baihui Wang

**Affiliations:** 1 School of Physical Education, Yanshan University, Qinhuangdao 066004, China; 2 School of Sports Training and Science, Tianjin University of Sport, Tianjin 300211, China; 3 School of Sport and Leisure, Sichuan Tourism University, Chengdu 610100, China

**Keywords:** Tai chi, sleep disorders, sleep quality, depression, anxiety, meta-analysis, RCT

## Abstract

To systematically review the effects of Tai chi on sleep quality, depression, and anxiety in patients with insomnia. The electronic databases including PubMed, Cochrane Library, Web of Science, Embase, China National Knowledge Infrastructure (CNKI), WanFang Data, Chinese Biomedical Literature Database (CBM), and VIP Database for Chinese Technical Periodicals (VIP) were retrieved and screened by computer. Randomized controlled trials (RCT) on patients with insomnia who practiced Tai chi were collected, and the RCT risk of bias assessment criteria was used to evaluate the methodological quality of the included studies. The combined effect size was expressed as the weighted mean difference (WMD), with a confidence interval of 95% (CI). Review Manager 5.4 and Stata16.0 were used for heterogeneity analysis and sensitivity analysis. Tai chi reduced the patients’ Pittsburgh sleep quality index (PSQI) score (WMD = −1.75, 95% CI: −1.88, −1.62, *p* < 0.001); Hamilton depression scale (HAMD) score (WMD = −5.08, 95% CI: −5.46, −4.69, *p* < 0.001), Hamilton anxiety scale (HAMA) score (WMD = −2.18, 95% CI: −2.98, −1.37, *p* < 0.001), and self-rating anxiety scale (SAS) score (WMD = −7.01, 95% CI: −7.72, −6.29, *p* < 0.001). Tai chi exercise has a good preventive and ameliorating effect on insomnia, which can relieve patients’ depression and anxiety, simultaneously enhancing various functions of the body. However, most of the included studies reported random assignment with some lack of specific descriptions, and the blinding of participants was difficult to achieve due to the nature of exercise, which may cause bias. Therefore, more high-quality, multi-center, and bigger-sample studies need to be included in the future to further verify the results.

## 1. Introduction

Insomnia is a common sleep disorder characterized by difficulty in falling asleep, easy awakening, etc., which is also a subjective experience of unsatisfactory sleep quality that is caused by frequent and persistent difficulty in falling asleep or difficulty in maintaining sleep in the suitable sleep opportunity and environment [1,2]. It is also often accompanied by functional impairment in family, social, occupational, academic, or other important areas [3]. A survey shows that about 30% of people have insomnia symptoms [4]. Furthermore, 10% of these people have met the diagnostic criteria for sleep disorders in *The Diagnostic and Statistical Manual of Mental Disorders* (DSM-IV) [5,6]. Studies have shown that long-term insomnia not only affects patients’ life and work but also leads to cognitive decline, anxiety, depression, and other mental diseases in severe cases, and even cardiovascular physical diseases [7,8]. At present, insomnia is still mainly treated with sedative-hypnotic drugs. In the guidelines for the diagnosis and treatment of insomnia, zolpidem, zopiclone, zaleplon, and other non-benzodiazepine drugs (NBZDs) are the drugs of first choice because of short half-life, low residual effect on the next day, effective treatment, and no serious adverse reactions [9,10]. However, long-term use of such drugs will still impair the patients’ next-day motor, executive, and neurocognitive functions [11]. In addition, the hypnotic effect may be reduced due to continuous use of such drugs [12].

Tai chi is a traditional health preservation method that integrates “daoyin” and “breathing” [13]. Combining “yi”, “qi”, and “gong” to regulate the physiological functions of the organs, tissues and cells of the whole body, it is carried out through muscle activation via orderly movement to suppress arousal levels, shorten the awakening cycle, and, thereby, promote sleep [14,15]. Its light and soft movement process includes orderly movement, posture control, and breathing control, which is equivalent to a physical and mental movement that simultaneously combines aerobic exercise, breath training, and balance training [13,14,15,16]. Therefore, Tai chi can play a role in calming the mind, promoting the homeostasis of the central nervous system, and helping improve sleep quality [17].

However, there were some problems with the randomized controlled trials on the application of Tai chi to insomnia patients at home and abroad, such as small sample size, nonstandard inclusion and exclusion criteria for test objects, and inconsistent efficacy indicators. At the same time, there was also a lack of meta-analysis or systematic evaluation of the efficacy of Tai chi on insomnia patients at home and abroad. Therefore, this study adopted the method of meta-analysis to evaluate the effect of Tai chi intervention on insomnia and provided evidence based on Tai chi treatment of insomnia, so as to promote the widespread application of Tai chi in insomnia patients and to improve the quality of their sleep and life, relieve depression and anxiety, ease social and patient economic burden, and promote the development of Tai chi.

## 2. Methods

### 2.1. Literature Search

A systematic search of the literature prior to April 2022 was performed using databases (CNKI, Wanfang, CBM, CQVIP, Web of Science, Embase, PubMed, Cochrane Library, etc.) to collect randomized controlled trials (RCTs) on Tai chi intervention in patients with insomnia with search terms such as “Tai ji”, “Tai chi”, “Tai ji quan”, “sleep initiation and maintenance disorders”, “insomnia”, and “randomized controlled trial, RCT”(Box 1). The systematic review followed the Preferred Reporting Items for Systematic Reviews and Meta-Analysis (PRISMA) guidelines and was based on a registered review protocol accessible online (PROSPERO CRD42022345511). In addition, references of included studies were traced to obtain and supplement relevant references.

Box 1 **Retrieval strategy for PubMed.**

#1 “Sleep Initiation and Maintenance Disorders”[Mesh]
#2 Disorders of Initiating AND Maintaining Sleep OR DIMS Disorders of Initiating AND Maintaining Sleep OR Early Awakening OR Awakening, Early OR Nonorganic Insomnia OR Insomnia, Nonorganic OR Primary Insomnia OR Insomnia, Primary OR Transient Insomnia OR Insomnia, Transient OR Rebound Insomnia OR Insomnia, Rebound OR Secondary Insomnia OR Insomnia, Secondary OR Sleep Initiation Dysfunction OR Dysfunction, Sleep Initiation OR Dysfunctions, Sleep Initiation OR Sleep Initiation Dysfunctions OR Sleeplessness OR Insomnia Disorder OR Insomnia Disorders OR Insomnia OR Insomnias OR Chronic Insomnia OR Insomnia, Chronic OR Psychophysiological Insomnia OR Insomnia, Psychophysiological
#3 #1 OR #2
#4 “Tai Ji” [Mesh]
#5 Tai-ji OR Tai Chi OR Chi, Tai OR Tai Ji Quan OR Ji Quan, Tai OR Quan, Tai Ji OR Taiji OR Taijiquan OR T’ai Chi OR Tai Chi Chuan
#6 #4 OR #5
#7 randomized controlled trial [Publication Type] OR randomized OR placebo
#8 #3 AND #6 AND #7

### 2.2. Literature Inclusion and Exclusion Criteria

Document inclusion, exclusion, retrieval, and screening criteria were developed according to the PRISMA statement [18].

Inclusion criteria: (1) All included studies were RCTs. (2) There was no significant difference between the experimental group and the control group before the experiment. (3) The intervention group performed Tai chi, while the control group were subject to acupuncture, regular exercise, blank control, and other intervention methods different from Tai chi. (4) The Pittsburgh sleep quality index was used to screen eligible insomniacs. The outcome index was one of the following: PSQI index, HAMD index, HAMA index, or SAS index.

Exclusion criteria: (1) The outcome index did not meet the requirements, including animal experiments, systematic evaluation, or reviews. (2) Interventions did not match. (3) Non-randomized controlled trials. (4) Duplicate published literature. (5) The full text was not available. (6) Articles did not match the research content.

### 2.3. Data Extraction and Quality Assessment

The literature that met the inclusion criteria were read and evaluated, and their data were extracted, excluding the obviously unqualified articles. The extracted content of the literature included first author, publication year, country (region), type of research object, sample size, age, intervention measures (time, frequency, method), and outcome indicators.

The Cochrane risk assessment was used to evaluate the methodological quality of the included literature into 3 grades from high to low: high quality (5 scores and above), medium quality (3–4 scores), and low quality (2 scores and below), and to score the quality of the included literature according to its 3 levels of risk (low risk, high risk, and unclear).

### 2.4. Statistical Analysis

The outcome indicators of this study were continuous variable type data. Review Manage 5.4.1 was performed to make a meta-analysis of literature screening chart and a Cochrane bias risk assessment diagram. The statistical analysis was performed using Review Manage 5.4.1 and Stata 16.0 software. The combined effect was expressed by weighted mean difference (WMD) and 95% confidence interval (CI). *p* < 0.05 for the outcome indicated statistical significance; heterogeneity was evaluated using X-square (X^2^) and I-square (I^2^), with *p* and I^2^ as indexes to evaluate the size of heterogeneity. If I^2^ ≤ 50%, *p* ≥ 0.1, then the statistical heterogeneity among the studies was small, and a fixed-effects model was used for analysis. If I^2^ > 50%, *p* < 0.1, it meant that the heterogeneity was obvious, and a random-effects model was used to combine the effect sizes of the results, with the source of heterogeneity being analyzed through sensitivity analysis and meta-regression. According to the reference for effect size proposed by Cohen, an absolute value of the effect size of less than or equal to 0.2 indicated that the effect was small, 0.2 to 0.8 indicated medium effects, and more than or equal to 0.8 indicated large effects [19].

## 3. Results

### 3.1. Literature Search

**Figure 1:**
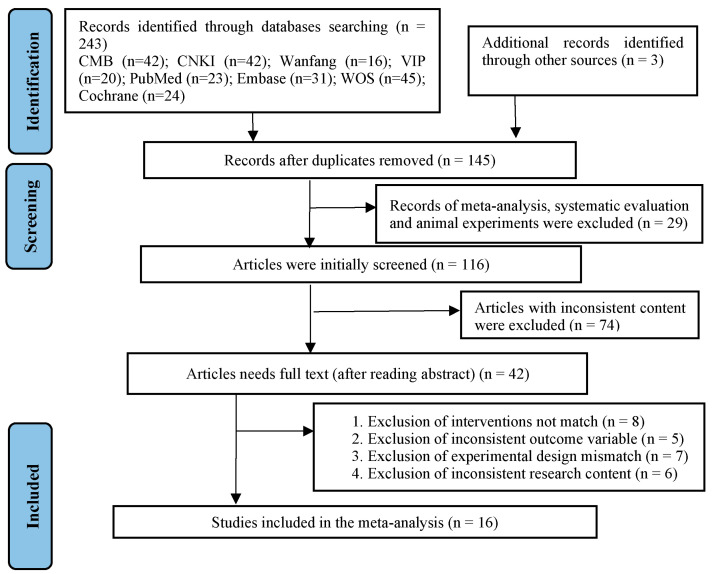
Flow of literature search and selection process.

Two researchers (JQ.Y and M.Y) searched and screened the database according to title, abstract, and full text. Altogether, 246 articles were identified, 243 by searching in various databases and 3 supplementary articles through manual retrieval. After importing the literature management software EndNote X9 to remove duplicate literature, a total of 145 articles were included. After reading the literature titles and abstracts, 103 irrelevant articles were excluded, leaving 42 articles, and after further reading the full text, 26 articles were excluded. Therefore, finally 16 articles on RCTs were included in meta-analysis (Figure 1). Table 1 displays the characteristics of the included studies.

### 3.2. Study Characteristics

The study included 16 articles from 5 countries, among them, 12 from China (Hong Kong), 3 from the United States, and 1 from Japan. The basic characteristics of the included studies are shown in table. In all studies, the mean total PSQI score at baseline was larger than or equal to 5. In addition to insomnia, some patients also suffer from other diseases, such as depression, anxiety, breast cancer, coronary heart disease, etc. This meta-analysis included 1547 patients, 781 of whom were assigned to the intervention group and 766 to the control group. The average age of the included patients was 54.22 years. Six articles [23,27,32,33,34,35] did not describe gender. In other included articles, 385 male patients and 517 female patients were found.

The studies included in this paper all used Tai chi as an intervention. Furthermore, 14 studies [20,21,22,23,24,25,26,27,28,29,30,32,33,35] described the style and routines of Tai chi, of which 7 studies [20,21,22,24,25,27,33] used 24-style Tai chi, 1 study [23] used Chen-style Tai chi, 1 study [26] used 8-style Tai chi, and 5 studies [28,29,30,32,35] used Yang-style Tai chi. As for the method of Tai chi practice, nine studies [20,21,23,26,29,30,31,32,33] were by means of expert teaching, five studies [22,24,25,27,35] were by independent practice, and other studies [28,34] did not elucidate on this. In terms of the intervention frequency and intervention time, the longest intervention was 25 weeks, and the shortest intervention was 2 weeks. The intervention frequency was mostly three times a week, and the intervention time was mostly 40–60 min.

The control group included in this review was divided into the non-treatment group and the treatment group, wherein, the non-treatment group was without any intervention. The treatment group included acupuncture, sports training, drug treatment, etc. Two studies [20,21] treated patients in the control group through acupuncture, six studies [24,27,30,32,33,35] by routine exercise, two studies [31,34] by health education, and two studies without intervention as a blank control group; the other six studies employed routine nursing [22], drug therapy [28], cognitive training [23], and psychotherapy [29].

### 3.3. Quality Evaluation

**Figure 2:**
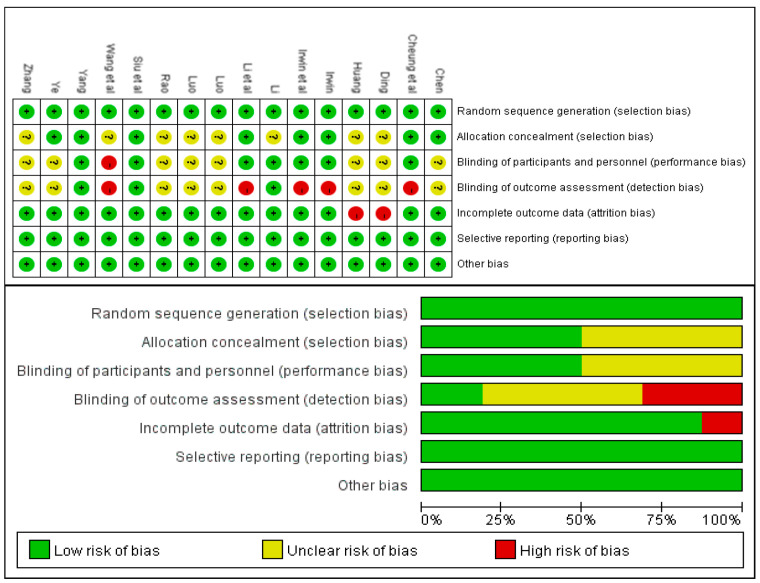
Included literature quality evaluation [20,21,22,23,24,25,26,27,28,29,30,31,32,33,34,35].

The methodological quality of the included studies was evaluated according to the Cochrane Version 5.1.0 tool. See Figure 2 for the included studies’ bias risk summary chart and the bias risk assessment chart. All the 16 included RCT studies [20,21,22,23,24,25,26,27,28,29,30,31,32,33,34,35] described the method of generating a random sequence. Only eight research reports used allocation concealment [22,27,29,30,31,32,33,34], and the other eight research reports did not elaborate on the allocation scheme [20,21,23,24,25,26,28,35]. Three studies [26,27,32] carried out the double-blind method, four studies [30,31,33,34] carried out a single-blind method, and other studies did not elaborate on this aspect. All study outcome indicators were complete, and there was no selective reporting.

### 3.4. Grade Evidence Quality Rating

**Figure 3:**
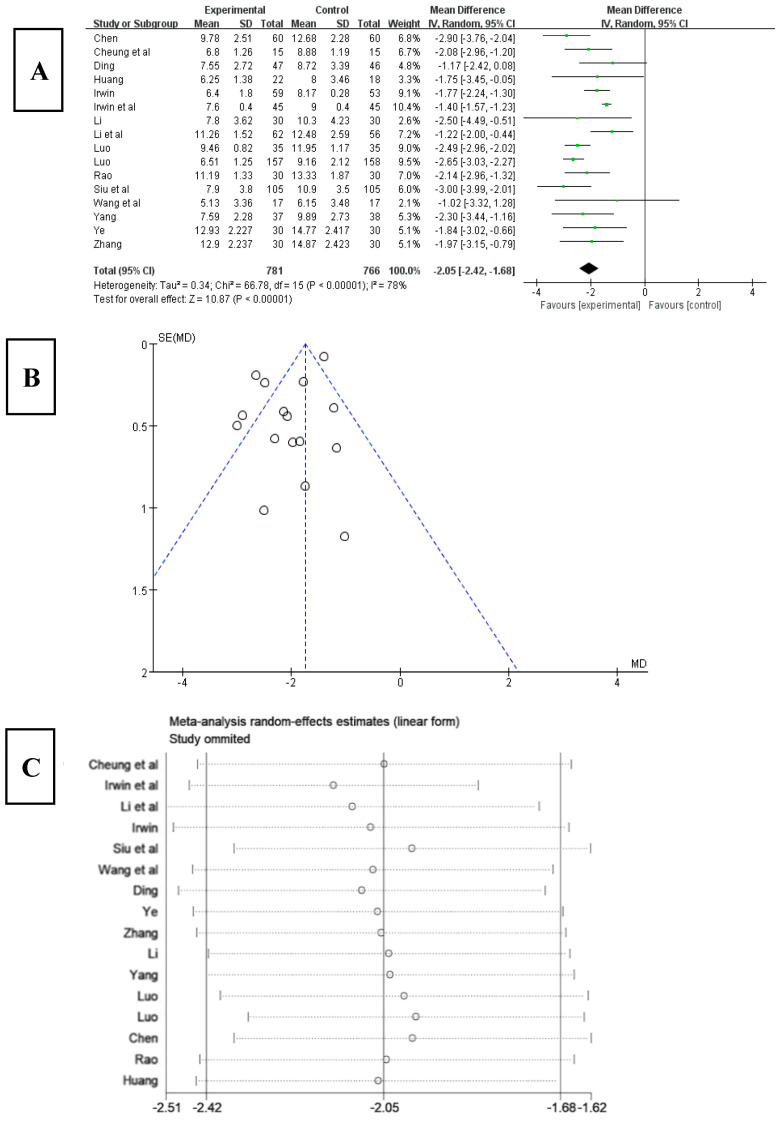
(**A**) Forest plot of PSQI effect size. (**B**) PSQI meta-analysis funnel chart. (**C**) Sensitivity analysis of PSQI effect size [20,21,22,23,24,25,26,27,28,29,30,31,32,33,34,35].

The GRADE grading system was used to evaluate the quality of evidence of outcome indicators, and the quality of evidence was divided into four levels: high, moderate, low, and very low. The default evidence quality of an RCT is high, which mainly evaluates the evidence quality of the outcome indicators from the five downgrade factors of research bias risk, inconsistency, indirectness, imprecision, and publication bias [36]; see Figure 3 for details.

### 3.5. Effects of Tai Chi on the PSQI of Patients with Insomnia

The 16 included studies [20,21,22,23,24,25,26,27,28,29,30,31,32,33,34,35] all used PSQI to assess patients’ sleep quality. According to the analysis of the influence of Tai chi on the PSQI index (Figure 3A), the PSQI index decreased after Tai chi intervention; the difference was statistically significant (WMD = −2.05, 95% CI: −2.42, −1.68, *p* < 0.001), and the results for heterogeneity among studies were Q = 66.78, df = 15, and I^2^ = 78% (*p* < 0.001). The results of funnel chart analysis of PSQI as an outcome indicator (Figure 3B) showed that the articles included in the study were symmetrical, but five articles appeared outside the funnel chart, suggesting that there may be publication bias.

In order to explore the source of heterogeneity, the included studies were respectively excluded using sensitivity analysis in the overall study to assess the impact of each study on the PSQI index effect size. The results (Figure 3C) suggested that there was not much heterogeneity among the included studies and that excluding a certain article had little effect on the PSQI index effect size. Therefore, the results of the meta-analysis were relatively stable.

To further explore the source of heterogeneity, a meta-regression analysis was conducted from the aspects of intervention year, intervention time, intervention frequency, sample size, article quality, and average age. To avoid false-positive results, at least 10 studies were required for each covariate. Therefore, heterogeneity was explored using univariate meta-regression analysis. From the results shown in Table 2, intervention frequency, sample size, article quality, and average age had no significant effect on heterogeneity (*p* > 0.05), while the intervention time had a relatively significant effect on heterogeneity (*p* < 0.05) that was statistically significant.

### 3.6. The Influence of Tai Chi on Various Indicators in PSQI

#### 3.6.1. Effects of Tai Chi on Sleep Quality in PSQI

**Figure 4:**
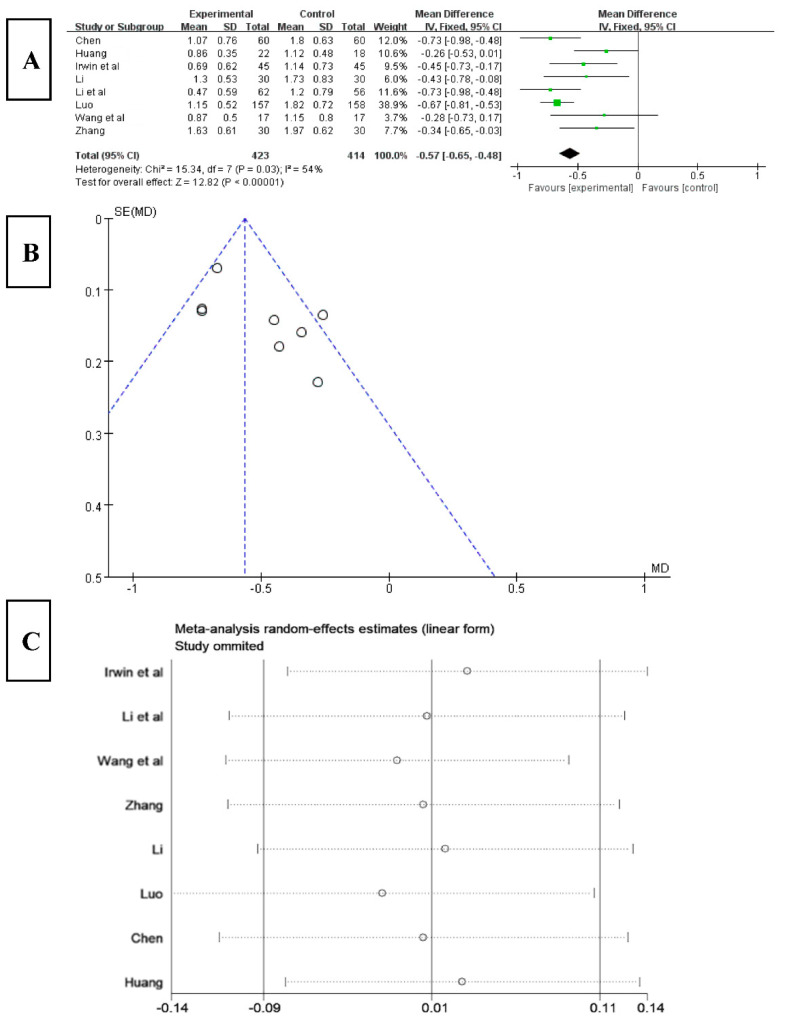
(**A**) Forest plot of sleep quality effect size. (**B**) Funnel diagram of sleep quality meta-analysis. (**C**) Sensitivity analysis of sleep quality effect size [22,24,25,26,28,33,34,35].

Among the 16 included studies, 8 studies analyzed sleep quality using the PSQI index [22,24,25,26,28,33,34,35]. The results (Figure 4A) showed that the score for sleep quality obviously decreased after Tai chi intervention (WMD = −0.57, 95% CI: −0.65, −0.48, *p* = 0.03), and it was statistically significant compared with the control group. The results of the heterogeneity test were Q = 15.34, df = 7 (*p* = 0.03), and I^2^ = 54%, indicating that there was a high degree of heterogeneity. Therefore, a random-effects model should be selected for meta-analysis and the source of heterogeneity should be explored. The results of funnel chart analysis of sleep quality as an outcome indicator (Figure 4B) showed that the articles included in the study were asymmetric and that there may be publication bias.

To explore the source of heterogeneity, sensitivity analysis was used in the overall study to exclude the included studies one by one, assessing the effect of each study on the sleep quality effect size. The results (Figure 4C) showed that, after excluding the study of Huang [24], the heterogeneity of the remaining research studies was reduced, Q = 9.66, df = 6 (*p* = 0.14), and I^2^ = 38%, and the 95% CI of the pooling effect magnitude −0.57 was [−0.70, −0.44], *p* < 0.01, indicating that Tai chi intervention had an impact on the PSQI of insomnia patients, improving their sleep quality.

#### 3.6.2. Effect of Tai Chi on Sleep Latency in PSQI

**Figure 5:**
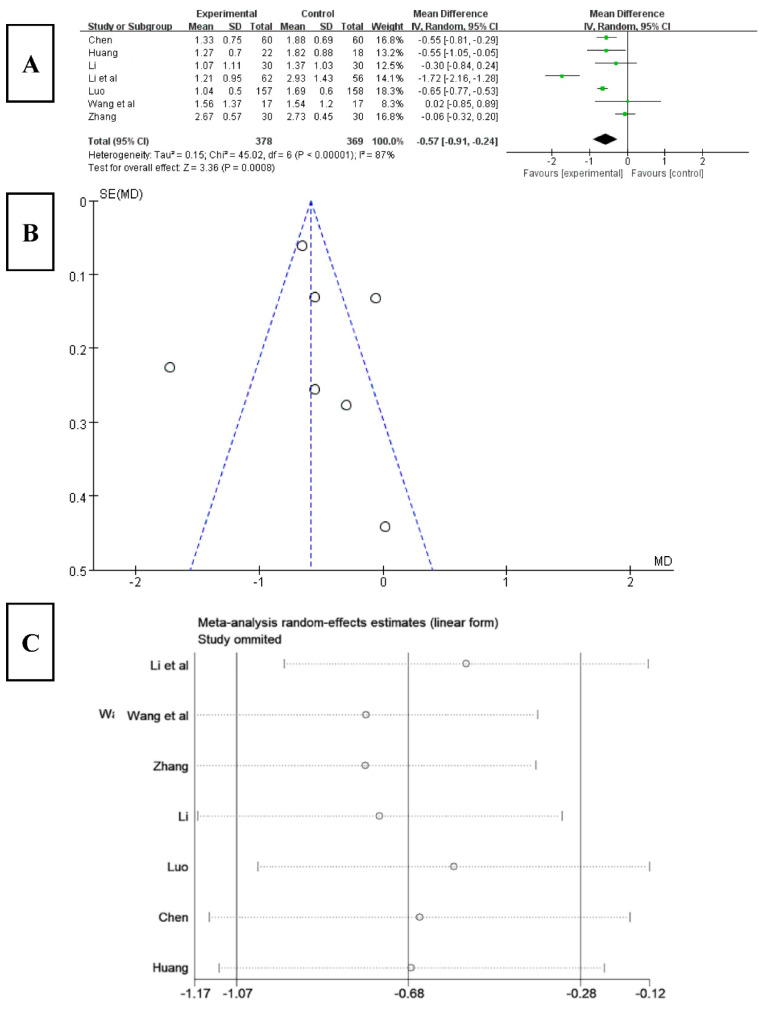
(**A**) Forest plot of sleep latency effect size. (**B**) Funnel chart of meta-analysis of sleep time. (**C**) Sensitivity analysis of effect size of sleep latency [22,24,25,26,28,33,35].

Among the 16 included studies, 7 studies analyzed sleep latency using the PSQI index [22,24,25,26,28,32,35]. The results (Figure 5A) showed that the score for sleep latency obviously decreased after Tai chi intervention (WMD = −0.57, 95% CI: −0.91, −0.24, *p* < 0.001), and it was statistically significant compared with the control group. The results of the heterogeneity test were Q = 45.02, df = 6 (*p* < 0.01), and I^2^ = 87%, indicating that there was a high degree of heterogeneity. Therefore, a random-effects model should be selected for meta-analysis. According to the funnel chart analysis of sleep latency (Figure 5B), the results showed that two articles included in the study were outside the funnel chart, showing an overall asymmetry, where there may be publication bias.

To explore the source of heterogeneity, a sensitivity analysis was used in the overall study to exclude the included studies one by one, and the effect of each study on the effect size of sleep latency was assessed. The results (Figure 5C) showed that after excluding the studies of Li [33] and Zhang [28], the heterogeneity of the remaining research studies was reduced, with Q = 3.96, df = 4 (*p* = 0.41), and I^2^ = 0%, and the 95% CI of the pooling effect magnitude −0.61 was [−0.71, −0.50], *p* < 0.001, indicating that Tai chi intervention had an impact on patients’ sleep latency, effectively reducing their sleep latency.

#### 3.6.3. Effects of Tai Chi on Sleep Time in PSQI

**Figure 6:**
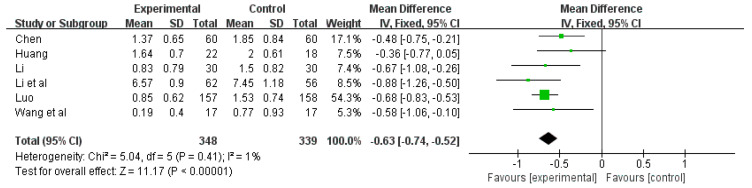
Forest plot of sleep time effect size [22,24,25,26,33,35].

Among the 16 included studies, 6 studies analyzed sleep time using the PSQI index [24,25,26,33,34,35]. There was no significant heterogeneity among the included studies (Q = 5.11, df = 5, *p* = 0.41, I^2^ = 1%); therefore, the fixed-effects model was used for meta-analysis. The results of meta-analysis (Figure 6) showed that Tai chi intervention was significantly superior to the control group in the perspective of improving sleep time in the PSQI index (WMD = −0.63, 95% CI: −0.74, −0.52, *p* < 0.001). It indicated that Tai chi intervention had an effect on the sleep time of patients, increasing their sleep time.

#### 3.6.4. Effects of Tai Chi on Sleep Efficiency in PSQI

**Figure 7:**
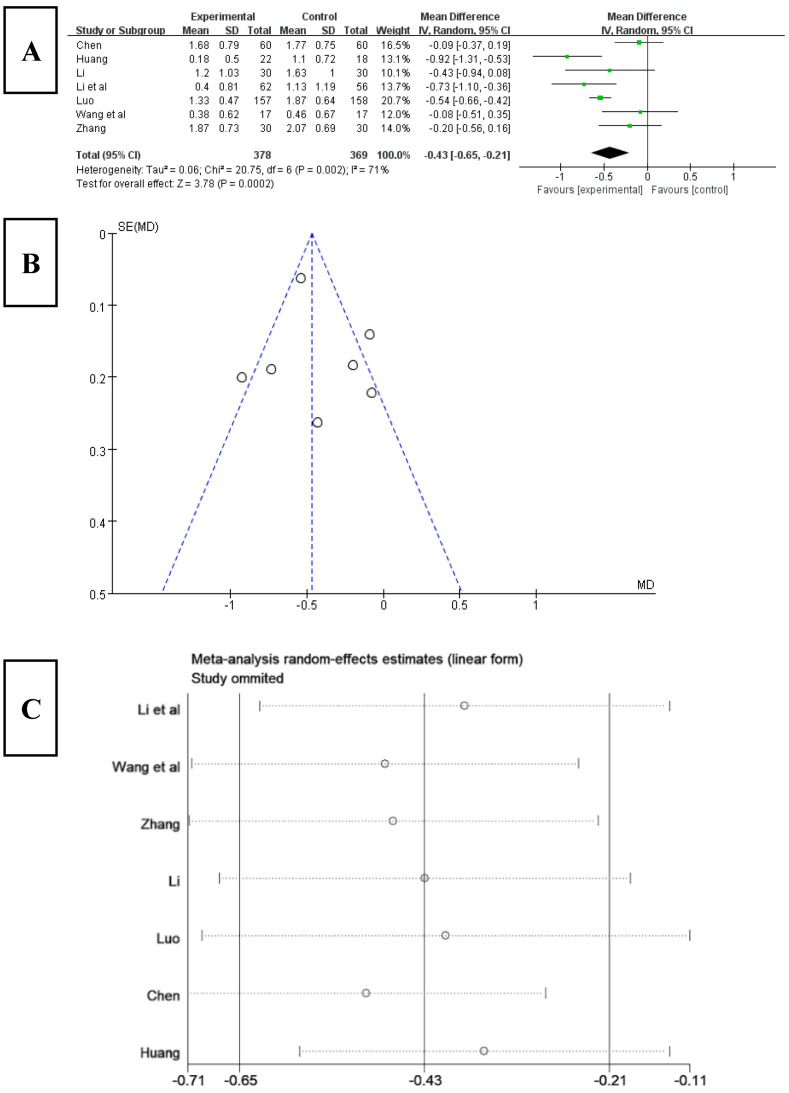
(**A**) Forest plot of sleep efficiency effect size. (**B**) Funnel chart of meta-analysis on sleep efficiency. (**C**) Sensitivity analysis of sleep efficiency effect size [22,24,25,26,28,33,35].

Among the 16 included studies, 7 studies analyzed sleep efficiency using the PSQI index [22,24,25,26,28,33,35]. The results (Figure 7A) showed that the score for sleep efficiency significantly decreased after Tai chi intervention (WMD = −0.43, 95% CI: −0.65, −0.21, *p* < 0.001), and it was statistically significant compared with the control group. The results of the heterogeneity test were Q = 20.75, df = 6 (*p* < 0.01), and I^2^ = 71%, indicating that there was a high degree of heterogeneity. Therefore, a random-effects model should be selected for meta-analysis. The results of funnel chart analysis of sleep efficiency as an outcome indicator (Figure 7B) showed that two articles included in the study were outside the funnel chart, showing overall symmetry.

To explore the source of heterogeneity, a sensitivity analysis was used to exclude the included studies one by one in the overall study to assess the effect of each study on the effect size of sleep efficiency. The results (Figure 7C) showed that, after excluding the studies of Li [34], Luo [25], and Huang [24], the heterogeneity of the remaining research studies was reduced, Q = 1.49, df = 3, and I^2^ = 0%, and the 95% CI of the pooling effect magnitude −0.16 was [−0.34, 0.02], *p* = 0.69 > 0.05, indicating that Tai chi intervention had an impact on the results of sleep efficiency in patients with insomnia, improving the sleep efficiency of patients.

#### 3.6.5. Effects of Tai Chi on Sleep Disorders in PSQI

**Figure 8:**
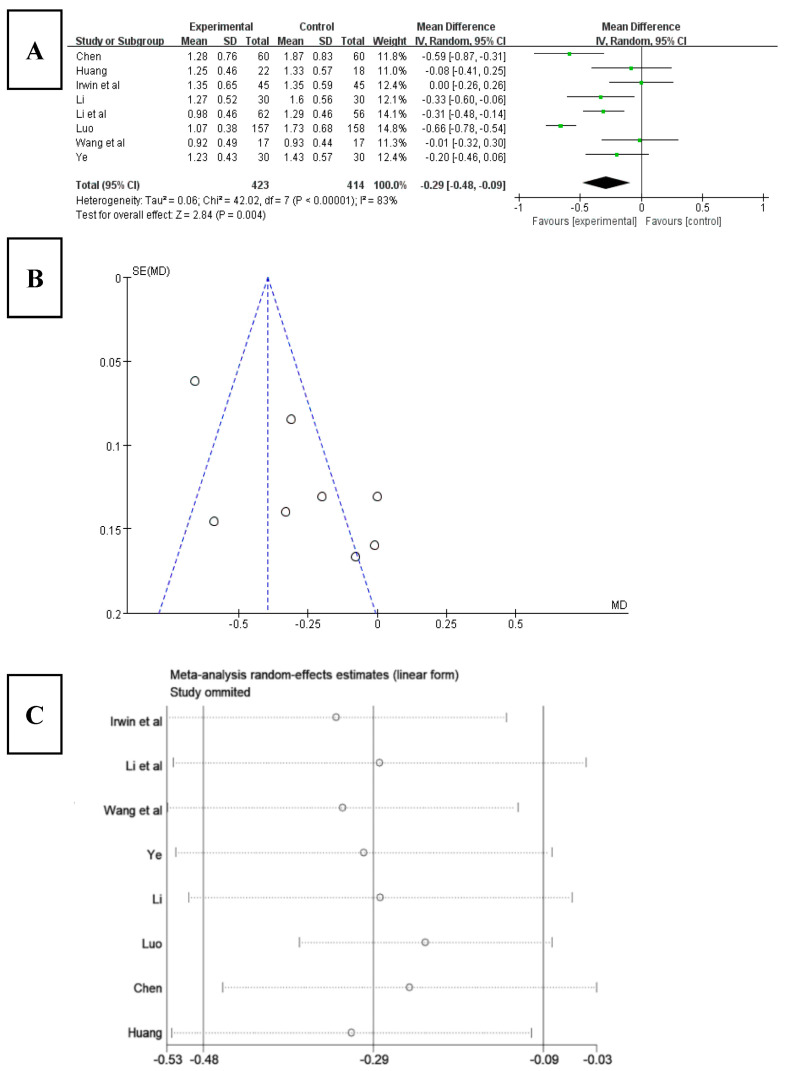
(**A**) Forest plot of sleep disorder effect size. (**B**) Funnel diagram of meta-analysis of sleep disorders. (**C**) Sensitivity analysis of sleep disorder effect size [22,24,25,26,29,33,34,35].

Among the 16 included studies, 8 studies analyzed sleep disorders using the PSQI index [22,24,25,26,29,33,34,35]. The results (Figure 8A) showed that the score for sleep disorder significantly decreased after Tai chi intervention (WMD = −0.29, 95% CI: −0.48, −0.09, *p* < 0.001), and it was statistically significant compared with the control group. The results of the heterogeneity test were Q = 42.02, df = 7 (*p* < 0.01), and I^2^ = 83%, indicating that there was a high degree of heterogeneity. Therefore, a random-effects model should be selected for meta-analysis. The results of funnel chart analysis of sleep disorder as an outcome index (Figure 8B) showed that three articles included in the study fell outside the funnel chart, showing overall asymmetry and possible publication bias.

To explore the source of heterogeneity, a sensitivity analysis was used to exclude the included studies individually from the overall study to assess the effect of each study on sleep disturbance effect size. The results (Figure 8C) showed that, after excluding the studies of Irwin [34] and Luo [25], the heterogeneity of the remaining research studies was reduced, Q = 9.48, df = 5, *p* = 0.09, and I^2^ = 47%, and the 95% CI of the pooling effect magnitude −0.27 was [−0.41, −0.12], *p* < 0.001, indicating that Tai chi intervention had an impact on sleep disorders in patients with insomnia, which alleviated patients’ sleep disorders to a certain degree.

#### 3.6.6. Effects of Tai Chi on Hypnotics in PSQI

**Figure 9:**
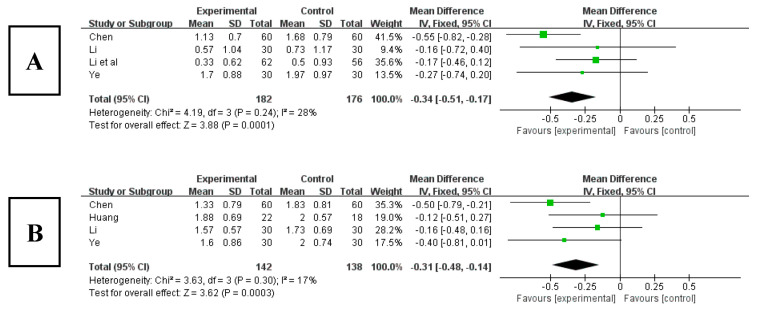
(**A**) Forest plot of effect size of hypnotics. (**B**) Forest plot of daytime dysfunction effect size [22,24,26,29,33].

Among the 16 included studies, there were 4 studies that analyzed hypnotics using the PSQI index [22,26,29,33]. The results (Figure 9A) showed that the score for use of hypnotics significantly decreased after Tai chi intervention (WMD = −0.34, 95% CI: −0.51, −0.17, *p* < 0.001), and it was statistically significant compared with the control group. The results of the heterogeneity test were Q = 4.19, df = 3 (*p* = 0.24), and I^2^ = 0%, indicating that there was no heterogeneity. Therefore, it suggested that Tai chi intervention had an effect on reducing the use of hypnotics of patients.

#### 3.6.7. Effects of Tai Chi on Daytime Dysfunction in PSQI

Among the 16 included studies, 4 studies analyzed daytime dysfunction using the PSQI index [22,24,26,29]. The results (Figure 9B) showed that the score for daytime dysfunction significantly decreased after Tai chi intervention (WMD = −0.31, 95% CI: −0.48, −0.14, *p* < 0.001), and it was statistically significant compared with the control group. The results of the heterogeneity test were Q = 3.63, df = 3 (*p* = 0.30), and I^2^ = 0%, indicating that there was no heterogeneity. Therefore, it suggested that Tai chi intervention can effectively relieve patients’ daytime dysfunction.

### 3.7. Effects of Tai Chi on the Mood of Patients with Sleep Disorders

#### 3.7.1. Effects of Tai Chi on HAMD in Patients with Sleep Disorders

**Figure 10:**
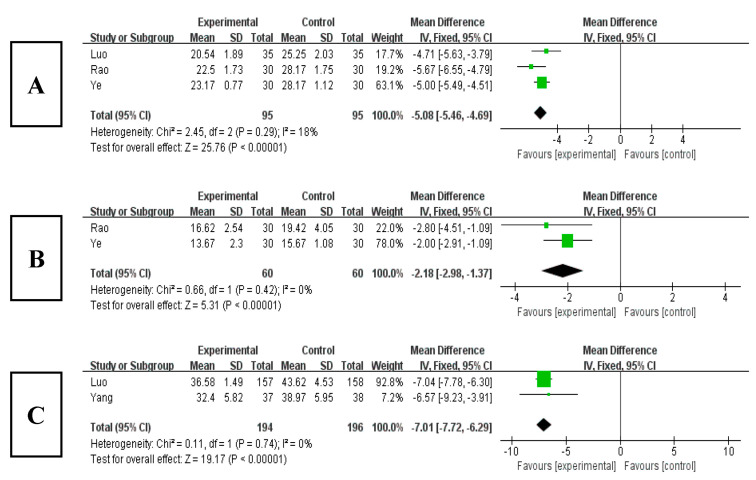
(**A**) Forest plot of HAMD effect size. (**B**) Forest plot of HAMA effect size. (**C**) Forest plot of SAS effect size [20,21,25,27,29].

Among the 16 included studies, 3 studies analyzed the HAMD index [20,21,29]. The results (Figure 10A) showed that the HAMD score obviously decreased after Tai chi intervention (WMD = −5.08, 95% CI: −5.46, −4.69, *p* < 0.001), and it was statistically significant compared with the control group. The results of the heterogeneity test were Q = 2.45, df = 3 (*p* = 0.29), and I^2^ = 18%, indicating that there was no heterogeneity. Therefore, it showed that Tai chi intervention can improve patients’ depressed mood.

#### 3.7.2. Effects of Tai Chi on HAMA in Patients with Sleep Disorders

Among the 16 included studies, 2 studies analyzed the HAMA index [21,29]. The results (Figure 10B) showed that the HAMA score obviously decreased after Tai chi intervention (WMD = −2.18, 95% CI: −2.98, −1.37, *p* < 0.001), and it was statistically significant compared with the control group. The results of the heterogeneity test were Q = 0.66, df = 1(*p* = 0.42), and I^2^ = 0%, indicating that there was no heterogeneity. Therefore, it showed that Tai chi intervention can effectively relieve patients’ anxiety state.

#### 3.7.3. Effects of Tai Chi on SAS in Patients with Sleep Disorders

Among the 16 included studies, 2 studies analyzed the SAS index [25,27]. The results (Figure 10C) showed that the SAS score obviously decreased after Tai chi intervention (WMD = −7.01, 95% CI: −7.72, −6.29, *p* < 0.001), and it was statistically significant compared with the control group. The results of the heterogeneity test were Q = 0.11, df = 1 (*p* = 0.74), and I^2^ = 0%, indicating that there was no heterogeneity. Therefore, it showed that Tai chi intervention can effectively relieve patients’ subjective anxiety.

### 3.8. GRADE Evidence Quality Rating

GRADE evidence quality evaluation was carried out for all included outcome indicators, among which PSQI was very-low-quality evidence and HAMD, HAMA, and SAS were moderate quality evidence, as shown in Table 3.

## 4. Discussion

Insomnia is a common sleep disorder, with sufferers facing chronic and prolonged sleep problems. Research showed that about half of patients with severe insomnia may experience symptoms for more than 10 years [37]. Epidemiological studies have found that the onset age of insomnia is decreasing year by year, and its incidence rate is growing [38]. With the gradual deepening of the research on insomnia at home and abroad, the treatment with drugs tends to be individualized, emphasizing the position of non-drug therapy in the treatment of insomnia, and the treatment is also increasingly improved for the elderly, pregnant people, children, or other people with chronic diseases [39,40]. However, the non-drug treatment of insomnia still has poor patient compliance, low performance for CBTI, and incomplete treatment systems, which need to be further improved. In contrast, Tai chi, which is simple and easy to learn, is not restricted by venue or equipment and is safe and cost-effective. However, there are few relevant studies on its use in the treatment of insomnia at home and abroad. Therefore, this study discussed the improvement effect of Tai chi on insomnia through a meta-analysis, with a view to popularizing this simple and efficient method, so as to improve the sleep quality of insomnia patients, relieve depression and anxiety, and reduce the cost of treatment.

A meta-analysis of the intervention of Tai chi on the sleep quality of the elderly has verified the overall effect of Tai chi exercise on the PSQI score through the data of 381 subjects in five studies. The results showed that Tai chi could significantly reduce the PSQI of patients. At the same time, further analysis of PSQI dimensions found that Tai chi had a good effect on improving sleep quality and daytime dysfunction and had a moderate effect on sleep latency and sleep time [41]. Another meta-analysis comprehensively analyzed the intervention effect of various types of Tai chi. Briefly, 24-style, 8-style, and Yang-style Tai chi had a significant impact on sleep quality [42]. Therefore, through meta-analysis, this study not only discussed the intervention effect of Tai chi on PSQI and its various dimensions in patients with insomnia, but also observed patients’ depression and anxiety and diagnosed and evaluated the insomnia, depression, and anxiety of patients, so as to evaluate the efficacy of Tai chi.

Studies have shown that exercise, pro-inflammatory factors, and sleep are inseparable. Tai chi is an aerobic exercise, which can improve and regulate pro-inflammatory factors. Inflammatory factors are not only involved in the immune response and inflammatory reaction but are also the pivotal factors between neural, endocrine, and immune networks, participating in sleep-to-wake rhythm regulation and improving sleep quality [43]. Other studies have shown that Tai chi can increase the body’s metabolic rate [44], enhance the blood supply to the core organs, improve sleep efficiency, promote deep sleep, and improve sleep quality [45].

Studies have found that about 20% of patients with insomnia will suffer depression, anxiety, and other unhealthy emotions, and, vice versa, excessive and long-term depression and anxiety will aggravate insomnia symptoms [46,47]. Tai chi emphasizes consciousness, breathing, and limb movements to perform moderate-intensity aerobic exercise in a gentle and slow way, in which the intensity is easy to control. It enhances the practitioners’ muscle strength and stimulates and strengthens breathing and cardiovascular system. In the meanwhile, Tai chi exercise can build up the practitioners’ spirit and will. Studies have shown that the brain of Tai chi practitioners can promote the body to produce catecholamines in a good state of awakening, which can make people happy, relieve excessive nervous system tension, and reduce stress, thereby eliminating and slowing down negativity, depression, anxiety, and other unhealthy emotions [48]. Further studies showed that long-term regular Tai chi exercise can stimulate the nervous system of the body to produce micro-electrical stimulation and promote the secretion of dopamine by substantia nigra cells [44], thereby reducing the influence of negative emotions such as depression and anxiety, hence reducing the symptoms of insomnia [45].

The results of this paper showed that Tai chi can effectively prevent and improve the symptoms of insomnia. The physiological mechanisms of Tai chi intervention include the following aspects: (1) Tai chi is a “complex” physical and mental exercise with both aerobic exercise and cognitive training components, bestowing the unique advantages of being simple, easy to learn, safe, and effective [49]. (2) Tai chi increases the blood flow rate and metabolic level of the body, nourishes neurotrophic factors and mRNA on the sleep nucleus, provides nutrition and neuroprotection to protect and support the different subgroups of neurons, and also plays a protective role of neurotransmitters in the sleep nucleus [50]. (3) Sleep disturbance is related to the pro-inflammatory state of the body [51]. Tai chi can reduce visceral fat content and the circulating number of pro-inflammatory monocytes, as well as increase the circulating number of regulatory T cells, thereby reducing inflammatory response and improving sleep quality [44,52].

This meta-analysis was conducted strictly in accordance with the PRISMA statement list, but there were still some limitations: (1) This study included only published studies, without unpublished studies, which may affect the comprehensiveness of the data to a certain extent. (2) Most of the studies reported random assignment, but some lacked specific description; in addition, the trial design may not have been rigorous, and, being an exercise intervention, it was difficult to achieve blinding of participants, which may cause some bias. (3) The number of included studies was limited, and more high-quality, multi-center, and large-sample studies need to be included in the future to further verify the results. In addition, GRADE evidence quality rating showed that HAMA, HAMD, and SAS were moderate, and PSQI evidence quality was very low, which suggests that the credibility of these outcome indicators needs to be strengthened, and the relevant results need to be treated with caution.

## 5. Conclusions

Tai chi can significantly enhance sleep quality, increase sleep time, reduce sleep latency, improve sleep efficiency, relieve sleep disorders and daytime dysfunction, as well as reduce the use of hypnotic drugs, which has a good prevention and improvement effect. At the same time, Tai chi can effectively reduce the negative emotions such as depression and anxiety in patients, thereby improving and alleviating the symptoms of insomnia.

## Figures and Tables

**Table 1:** Characteristics of the included studies.

Author	Years	Area	n (T/C)	Age (T/C)	Key Interventions		Cycle/Week	Outcomes	Quality
					T	C			
Luo [20]	2020	China	35/35	60.07 ± 4.01/60.03 ± 4.05	Tai chi	acupuncture	8/7	①②	4
Rao et al. [21]	2018	China	30/30	54.91+4.65/54.32+4.99	Tai chi	acupuncture	8/5	①②③	4
Chen et al. [22]	2017	China	60/60	61.14 ± 5.30/60.60 ± 6.23	Tai chi	routine care	12/3	①	5
Ding [23]	2020	China	47/46	60–80	Tai chi	cognition	12/3	①	3
Huang [24]	2016	China	22/18	18.68 ± 1.76/18.67 ± 1.50	Tai chi	exercise	24/2	①	3
Luo [25]	2021	China	157/158	18.58 ± 2.25	Tai chi	blank	8/3	①④	4
Li [26]	2020	China	30/30	79.13 ± 5.88/77.33 ± 6.05	Tai chi	blank	12/4	①	6
Yang [27]	2018	China	37/38	60–75	Tai chi	exercise	16/3	①④	7
Zhang [28]	2014	China	30/30	48.4 ± 6.7/48.9 ± 7.4	Tai chi	medical	2/7	①	4
Ye [29]	2012	China	30/30	56.37 ± 9.413/55.42 ± 9.76	Tai chi	psychotherapy	8/2	①②③	5
Cheung et al. [30]	2021	HK	15/15	61.11 ± 7.01/61.00 ± 12.12	Tai chi	exercise	12/2	①	6
Irwin et al. [31]	2008	USA	59/53	69.7 ± 6.1/69.8 ± 7.6	Tai chi	health education	16/3	①	6
Siu et al. [32]	2021	HK	105/105	66.5 ± 6.4/66.5 ± 6.4	Tai chi	exercise	12/3	①	7
Li et al. [33]	2004	USA	62/56	75.30 ± 7.8/75.45 ± 7.8	Tai chi	exercise	24/3	①	6
Irwin et al. [34]	2017	USA	45/45	59.6 ± 7.9/60.0 ± 9.3	Tai chi	health education	25/2	①	5
Wang et al. [35]	2010	Japan	17/17	50+	Tai chi	exercise	12/1	①	5

Note: T = experimental group; C = control group; ① PSQI = Pittsburgh sleep quality index; ② HAMD = Hamilton depression scale; ③ HAMA = Hamilton anxiety scale; ④ SAS = self-rating anxiety scale.

**Table 2:** Meta-regression analysis of heterogeneity factors affecting PSQI index effect size.

Study Characteristics	Regression Coefficient (β)	95% CI	*t* Value	*p* Value
intervention time	0.092	0.043~0.140	4.15	0.002
intervention frequency	−0.018	−0.044~0.008	−1.54	0.154
sample size	−0.001	−0.043~−0.04	−0.08	0.935
article quality	−0.321	−0.729~0.088	−1.75	0.111
average age	0.020	−0.013~0.052	1.36	0.204
_Cons	−2.041	3.516~−0.567	−3.09	0.012

**Table 3:** Evidence quality rating of outcome indicators.

Outcome Index	RCTs/ Number	Risk of Bias	Inconsistency	Indirectness	Inaccuracy	Publication Bias	Effect Size (95% CI)	Evidence Quality Grade
PSQI	16	serious ①	serious ②	no	no	existing	MD= −1.75 [−1.88, −1.62]	very low
HAMD	3	serious ①	no	no	no	no	MD = −5.08 [−5.46, −4.69]	moderate
HAMA	2	serious ①	no	no	no	no	MD = −2.18 [−2.98, −1.37]	moderate
SAS	2	serious ①	no	no	no	no	MD = −7.01 [−7.72, −6.29]	moderate

PSQI: Pittsburgh sleep quality index; HAMD: Hamilton depression scale; HAMA: Hamilton anxiety scale; SAS: Self-rating anxiety scale; CI: Confidence interval; MD: Mean difference. ① Lack of blindness and insufficient allocation concealment; ② I^2^ = 78%.

## Data Availability

The original contributions presented in this study are included in the article, and further inquiries can be directed to the corresponding author.

## References

[1] Chinese Sleep Research Society (2017). Guidelines for the diagnosis and treatment of insomnia in China. Natl. Med. J. China.

[2] Winkley K., Upsher R., Stahl D., Pollard D., Kasera A., Brennan A., Heller S., Ismail K. (2020). Psychological interventions to improve self-management of type 1 and type 2 diabetes: A systematic review. Health Technol. Assess..

[3] Lu L., Shen Y.C. (2017). Psychology.

[4] Haynes J., Talbert M., Fox S., Close E. (2018). Cognitive behavioral therapy in the treatment of insomnia. South. Med. J..

[5] Soldatos C.R., Allaert F.A., Ohta T., Dikeos D.G. (2005). How do individuals sleep around the world? Results from a single-day survey in ten countries. Sleep Med..

[6] Bolge S.C., Doan J.F., Kannan H., Baran R.W. (2009). Association of insomnia with quality of life, work productivity, and activity impairment. Qual. Life Res..

[7] Meena S.K., Rita A. (2017). The effects of insomnia and sleep loss on cardiovascular disease. Sleep Med. Clin..

[8] Yuan R., Wang J., Guo L.L. (2015). Effects of sleep deprivation on coronary heart disease and progress in prevention and treatment of traditional Chinese medicine. Chin. J. Chin. Mater. Med..

[9] Zhang P., Li Y.P., Wu H.J., Zhao Z.X. (2018). Guidelines for the diagnosis and treatment of adult insomnia in China. Chin. J. Neur..

[10] He Q.N., Wang X.D., Huang M., Sun L. (2018). Research progress of drug therapy for chronic insomnia. Chin. J. Clin. Pharmacol..

[11] Sun Y.K., Shi L., Chen S.Q., Lin X., Lu L., Zhang X.J. (2017). Effects of sedative-hypnotic drug therapy on cognitive function in patients with insomnia. Chin. J. Nerv. Ment. Dis..

[12] Zhao F.Y., Duan Y.R., Yan H.X., Li A.Q., Hu Y., Zhang Z.Y., Xu H. (2016). Clinical evaluation of moxibustion combined with Taijiquan and Jacobson progressive muscle relaxation training on exercise-induced insomnia. J. Shenyang Sport Univ..

[13] Qiu H.F. (2009). Tai Chi.

[14] Krueger J.M., Obal F.J., Fang J., Kubota T., Taishi P. (2001). The role of cytokines in physiological sleep regulation. Ann. N. Y. Acad. Sci..

[15] Irwin M.R., Thompson J., Miller C., Gillin J.C., Ziegler M. (1999). Effects of sleep and sleepdeprivation on catecholamine and interleukin-2 levels in humans:clinical implications. J. Clin. Endocrinol. Metab..

[16] Wu H.Y., Shi J.M. (2015). Characteristics and production methods of exercise prescriptions for middle-aged and elderly people. Bull. Sport Sci. Tech..

[17] Song J., Lai H., Xie X.T., Huang J., Wu J.S. (2019). Research progress of Taijiquan intervention in elderly depressive disorder. Fujian J. Trad. Chin. Med..

[18] Liberati A., Altman D.G., Tetzlaff J., Mulrow C., Gotzsche P.C., Ioannidis J.P., Clarke M., Devereaux P.J., Kleijnen J., Moher D. (2009). The PRISMA statement for reporting systematic reviews and meta-analyses of studies that evaluate health care interventions: Explanation and elaboration. PLoS Med..

[19] Cohen J. (1988). Statistical Power Analysis for the Behavioral Sciences.

[20] Luo Y.H. (2020). Study on the improvement of sleep and mood in patients with depression-related insomnia by Taijiquan. BaoJian Wenhui.

[21] Rao T., Ni J.H. (2018). Effects of Taijiquan on sleep and mood in patients with depression-related insomnia. Chin. Prim. Health Care.

[22] Chen Y.Z., Wang X.Y., Zhang M. (2017). Effects of Taijiquan training on sleep quality of elderly patients in cardiology department. Chin. Manip. Qi Gong Ther..

[23] Ding Y.J. (2020). Research on the Intervention Effect of Taijiquan Exercise on Insomnia in the Elderly.

[24] Huang T. (2016). Effects of Taijiquan on the Secretion of Melatonin and Its Related Indexes in Sports Students with Sleep Disorders.

[25] Luo S.S. (2021). Research on the intervention effect of Taijiquan on college students’ sleep disorders. Chin. J. Conval. Med..

[26] Li Y.Q. (2020). A Randomized Controlled Experimental Study on the Effects of Eight-Style Taijiquan Exercise on the Mental Health of the Elderly in Nursing Homes.

[27] Yang X.W. (2018). Effects of Taijiquan on Sleep Quality in Elderly Patients with Essential Hypertension.

[28] Zhang Y.D. (2014). Observation on the effect of 24-style Taijiquan intervention on 60 cases of insomnia. Chin. Comm. Doc..

[29] Ye D. (2012). Research on TCM Syndrome of Insomnia in Hong Kong and Taijiquan Intervention.

[30] Cheung D.S.T., Takemura N., Lam T.C., Ho J.C.M., Deng W., Smith R., Yan Y., Lee A.W.M., Lin C.C. (2021). Feasibility of Aerobic Exercise and Tai-Chi Interventions in Advanced Lung Cancer Patients: A Randomized Controlled Trial. Integr. Cancer Ther..

[31] Irwin M.R., Olmstead R., Motivala S.J. (2008). Improving sleep quality in older adults with moderate sleep complaints: A randomized controlled trial of Tai Chi. Sleep.

[32] Siu P.M., Yu A.P., Tam B.T., Chin E.C., Yu D.S., Chung K.F., Hui S.S., Woo J., Fong D.Y., Lee P.H. (2021). Effects of Tai Chi or Exercise on Sleep in Older Adults With Insomnia: A Randomized Clinical Trial. JAMA. Netw. Open.

[33] Li F., Fisher K.J., Harmer P., Irbe D., Tearse R.G., Weimer C. (2004). Tai chi and self-rated quality of sleep and daytime sleepiness in older adults: A randomized controlled trial. J. Am. Geriatr. Soc..

[34] Irwin M.R., Olmstead R., Carrillo C., Sadeghi N., Nicassio P., Ganz P.A., Bower J.E. (2017). Tai Chi Chih Compared with Cognitive Behavioral Therapy for the Treatment of Insomnia in Survivors of Breast Cancer: A Randomized, Partially Blinded, Noninferiority Trial. J. Clin. Oncol..

[35] Wang W., Sawada M., Noriyama Y., Arita K., Ota T., Sadamatsu M., Kiyotou R., Hirai M., Kishimoto T. (2010). Tai Chi exercise versus rehabilitation for the elderly with cerebral vascular disorder: A single-blinded randomized controlled trial. Psychogeriatrics.

[36] Gordon G., Andrew D., Oxman E., Regina K., Gunn V., Jan B., Susan N., Yngve F., Paul G., Hans D. (2011). GRADE Guidelines: I Introduction–GRADE evidence summary table and results summary table. Chin. J. Evid. Based Med..

[37] American Academy of Sleep Medicine (2014). International Classification of Sleep Disorders.

[38] Shergis J.L., Ni X., Sarris J., Zhang A.L., Guo X., Xue C.C., Lu C., Hugel H. (2017). Ziziphus spinosa seeds for insomnia: A review of chemistry and psychopharmacology. Phytomedicine.

[39] Seow S.Y., Kwok K.F.V., Tay K.H., Chee W.S.A., Rawtaer I., Cheng Y., Tan Q.X., Tan S.M. (2022). Systematic Review of Clinical Practice Guidelines for Insomnia Disorder. J. Psychiatr. Pract..

[40] Liu S., Zhang B. (2017). Interpretation of Chinese guidelines for the diagnosis and treatment of insomnia. Chin. J. Contemp. Neurol. Neurosurg..

[41] Du S., Dong J., Zhang H., Jin S., Xu G., Liu Z., Chen L., Yin H., Sun Z. (2015). Taichi exercise for self-rated sleep quality in older people: A systematic review and meta-analysis. Int. J. Nurs. Stud..

[42] Li H., Chen J., Xu G., Duan Y., Huang D., Tang C., Liu J. (2020). The effect of Tai Chi for improving sleep quality: A systematic review and meta-analysis. J. Affect. Disord..

[43] Zhao C.L., Ouyang S.Y., Chen L.L., Su J., Li A.L. (2022). Effects of aerobic exercise on sleep quality, sleep structure and inflammatory factors in patients with primary insomnia. Chin. Nurs. Res..

[44] Nguyen M.H., Kruse A. (2012). A randomized controlled trial of Tai chi for balance, sleepquality and cognitive performance in elderly Vietnamese. Clin. Interv. Aging.

[45] Chang Y., Cheng S.Y., Lin M., Gao F.Y., Chao Y.F. (2010). The effectiveness of intradialytic legergometry exercise for improving sedentary life style and fatigue among patients withchronic kidney disease: A randomized clinical trial. Int. J. Nurs. Stud..

[46] Neu D., Mairesse O., Verbanck P., Le B.O. (2015). Slow wave sleep in the chronically fatigued: Power spectra distribution patterns in chronic fatigue syndrome and primary insomnia. Clin. Neurophysiol..

[47] Riemann D., Krone L.B., Wulff K., Nissen C. (2020). Sleep, insomnia, and depression. Neuropsychopharmacology.

[48] Li P.S., Wang L., Cao L. (2013). The influence of different types of sports on the mental health of college students. J. Shenyang Sport Univ..

[49] Wang Y.J., Xu S.L., Liu J. (2022). Research progress on brain effect mechanism of Tai Chi training. Rehabil. Med..

[50] Zhao Z.X. (2003). Clinical Sleep Disorders.

[51] Irwin M.R., Olmstead R., Breen E.C., Witarama T., Carrillo C., Sadeghi N., Arevalo J.M., Ma J., Nicassio P., Bootzin R. (2015). Cognitive behavioral therapy and tai chireverse cellular and genomic markers of inflammation in late-life insomnia: Arandomized controlled trial. Biol. Psychiatry.

[52] Huang L.Y., Yao L.Z., Huang T. Effects of Taijiquan Practice on Upstream and Downstream Factors of Melatonin. Proceedings of the 5th Shenjiang Int. Wushu Acad. Forum and Cai Longyun Wushu Thought Seminar.

